# Emollient therapy for preterm newborn infants – evidence from the developing world

**DOI:** 10.1186/1471-2458-13-S3-S31

**Published:** 2013-12-20

**Authors:** Rehana A Salam, Jai K Das, Gary L Darmstadt, Zulfiqar A Bhutta

**Affiliations:** 1 Division of Women & Child Health, The Aga Khan University, Karachi, Pakistan; 2 Global Development Division, Bill & Melinda Gates Foundation, Seattle, WA, USA; 3 Global Child Health and Policy, Centre for Global Child Health, The Hospital for Sick Children, Toronto, ON, Canada

## Abstract

**Introduction:**

Application of emollients is a widespread traditional newborn care practice in many low and middle-income countries (LMICs) and may have the potential to decrease infection and consequent mortality in preterm neonates.

**Methods:**

We systematically reviewed literature published up to December 2012 to identify studies describing the effectiveness of emollient therapy. We used a standardized abstraction and grading format to estimate the effect of emollient therapy by applying the standard Child Health Epidemiology Reference Group (CHERG) rules.

**Results:**

We included seven studies and one unpublished trial in this review. Topical emollient therapy significantly reduced neonatal mortality by 27% (RR: 0.73, 95% CI: 0.56, 0.94) and hospital acquired infection by 50% (RR: 0.50, 95% CI: 0.36, 0.71). There were significant increases in weight (g) (MD: 98.04, 95% CI: 42.64, 153.45) and weight gain (g/kg/day) (MD: 1.57, 95% CI: 0.79, 2.36), whereas the impacts were non-significant for length and head circumference.

**Conclusion:**

Emollient therapy is associated with improved weight gain, reduced risk of infection and associated newborn mortality in preterm neonates and is a potentially promising intervention for use in low resource settings. Large scale effectiveness trials are required to further assess the impact of this intervention.

## Introduction

Neonatal mortality is increasingly important in order to progress on the Millennium Development Goal (MDG) for child survival by 2015 and beyond, as 40% of under-five deaths occur in newborns and 14% of these are attributable to preterm birth complications [1]. Despite the progress in reducing under five mortality, advances in addressing low birth weight (LBW) and prematurity have been slow. Every year around 15 million babies are born preterm, mostly in Africa and South Asia, and over 1 million die due to complications of prematurity [2]. The mortality rate among preterm neonates less than 32 weeks gestational age in some developing countries is over 50% [3,4], and more than half of these deaths are attributable to vulnerability to infections [5]. Moreover, there has been an increase in preterm birth rates over the past 20 years [6].

Evidence suggests that compromised skin barrier function of preterm newborns serves as an important contributor towards increased susceptibility to infections, hence morbidity and mortality. The high prevalence of malnutrition and environmental load of pathogenic organisms in developing countries further enhances this vulnerability. Newborn oil massage is an intervention that has been a traditional practice in the Indian subcontinent for hundreds of years [7-9] and this acts by augmenting the mechanical barrier and also is a source of essential fatty acids like linoleic acid [10]. It provides a physical barrier to skin disruption, reduces microorganism invasion and consequently reduces hospital acquired infections [11]. It also reduces transepidermal water loss [12,13] and conserves heat and energy to promote growth [14].

Mustard oil is the most commonly used emollient, particularly in South Asia, but other natural plant oils like sunflower, sesame, coconut, olive, and soybean oils are also commonly available and used. Animal studies have raised concern that mustard oil is much less effective at promoting and maintaining skin integrity than other emollients [15]. More recently there have been studies suggesting the benefit of newborn massage on brain development, stress reduction and reduced risk of retinopathy of prematurity [16,17].

Despite the wide body of primary research on emollient therapy there are few syntheses of the existing data. A Cochrane review [18] on this subject only covered extremely low birth weight infants from High Income Countries (HIC). Given the potential for topical emollient therapy to prevent infection in preterm or low birth weight infants in low and middle income countries (LMICs), and the risks that this traditional practice may in fact be harmful, in this review, we have estimated the effect of topical emollient therapy on hospital acquired infection, growth, mortality and other health outcomes among hospitalized preterm infants. We have reviewed the available literature and evaluated the quality of included studies according to the Child Health Epidemiology Group (CHERG) adaptation of Grading of Recommendations, Assessments, Development and Education (GRADE) criteria [19].

## Methods

We systematically reviewed literature published up to December 2012 to identify studies describing the effectiveness of emollient therapy. Following CHERG Systematic Review Guidelines [19], we searched PubMed, Cochrane Libraries, Embase, and WHO Regional Databases to identify all published and unpublished clinical trials. Additional studies were identified by hand searching references from included studies. Search terms included combinations of emollient* OR “emollient therapy” OR “massage” OR skin care OR oils OR “plant oils” OR “vegetable oils” OR “mineral oil” OR sunflower OR safflower OR coconut OR soybean OR sesame OR massage AND preterm* OR “low birth weight” OR neonate* OR newborn OR LBW. No language or date restrictions were applied in the searches.

### Inclusion criteria

Studies evaluating prophylactic application of topical emollient to preterm neonates (< 37 weeks gestation) and starting within the first 96 hours after birth and continued for at least one week thereafter were included. Studies that included topical emollient versus routine skin care or one topical oil (or a combination of oils) versus another (or a combination of oils) were included. We excluded studies examining the impact of aquaphor, as these are mostly used in developed country settings, or those that were conducted in HIC.

### Abstraction, analysis and summary measure

We abstracted data describing study identifiers and context, study design and limitations, intervention specifics and outcome effects into a standardized abstraction form for studies that met the final inclusion criteria as detailed in the CHERG Systematic Review Guidelines [19]. Outcomes of interest included mortality, hospital acquired infections, skin condition, weight, length, head circumference and neuro-developmental outcomes. Each study was assessed and graded according to the CHERG adaptation of the GRADE technique [19].

### Quantitative data synthesis

We conducted a meta-analysis for individual studies and pooled statistics were reported as the relative risk (RR) for categorical variables and mean difference (MD) for continuous variables between the experimental and control groups with 95% confidence intervals (CI). Mantel–Haenszel pooled relative risk and corresponding 95% confidence interval (CI) were reported or the DerSimonian–Laird pooled relative risk and corresponding 95% CI where there was an unexplained heterogeneity. All analyses were conducted using the software Review Manager 5.1. Heterogeneity was quantified by Chi^2^ and I^2^, which can be interpreted as the percentage of the total variation between studies that is attributable to heterogeneity rather than to chance; a low p-value (less than 0.1) or a large chi-squared statistic relative to its degree of freedom and I^2^ values greater than 50% were taken as substantial and high heterogeneity. In situations of high heterogeneity, causes were explored by sensitivity analysis and random effect models were used.

We summarized the evidence by outcome, including qualitative assessments of study quality and quantitative measures, according to the standard guidelines. A grade of “high”, “moderate”, “low” and “very low” was used for grading the overall evidence indicating the strength of an effect on specific health outcome according to the CHERG Rules for Evidence Review [19].

## Results

**Figure 1:**
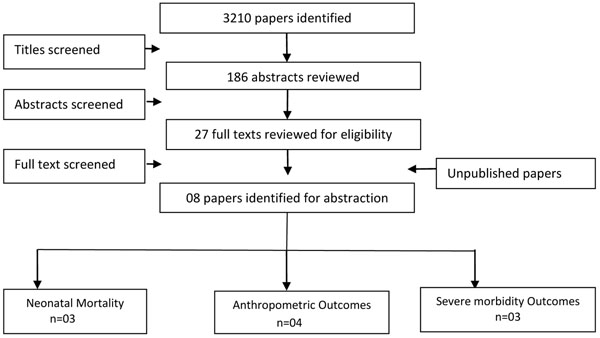
Search flow diagram

We identified 3210 titles from searches conducted in all databases. After screening titles and abstracts, we reviewed 27 papers for the identified outcome measures of interest of which seven [11,14,20-24] papers were selected for inclusion, which evaluated the impact of topical emollient therapy versus no intervention or control (Figure 1). We also included data from one unpublished trial [25]. Included studies used sunflower, coconut, soybean or mineral oil as emollients and all were conducted in developing countries. Two studies [11,21] included neonates < 33 weeks gestation, two studies [20,23] included neonates < 34 weeks gestation, three studies [14,22,25] included neonates <37 weeks gestation while one study [24] included neonates <35 weeks gestation. Three studies [11,20,21] identified gestational age according to the Dubowitz and Ballard, and on the basis of maternal dates while one study [25] estimated from ultrasound records and Ballard scores at admission, whereas the other studies have not reported on the method (Table 1).

**Table 1:** Characteristics of the included studies

Author	Country	Target population	Emollient	Dose and frequency	Follow up
Arora 2005 [14]	India	Neonates <37 weeks and <1500 g	Sunflower oil	4 times a day (10ml/kg/day)	28 days

Darmstadt 2004 [20]	Egypt	Neonates <34 weeks and <72hr old. Gestational age was determined with an average of gestational age values by last menstrual period, per the criteria of Ballard and Dubowitz	Sunflower oil	3 times a day for 14 days and then 2 times a day (12g/kg/day)	28 days

Darmstadt 2005 [11]	Bangladesh	Neonates <33 weeks and <72hr old. Gestational age was identified by doctors, according to the methods described by Dubowitz and Ballard, and on the basis of maternal dates (time from the first day of the last menstrual period); the average of the three measures was used	A. Sunflower seed oil B. Aquaphor	3 times a day for 14 days and then 2 times a day (12g/kg/day)	28 days or until discharge if <28 days

Darmstadt 2008 [21]	Bangladesh	Neonates <33 weeks and <72hr old. Gestational age was identified by doctors, according to the methods described by Dubowitz and Ballard, and on the basis of maternal dates (time from the first day of the last menstrual period); the average of the three measures was used	A. Sunflower seed oil B. Aquaphor	3 times a day for 14 days and then 2 times a day (12g/kg/day)	28 days or until discharge if <28 days

Kumar 2012 [24]	India	Neonates <35 weeks and <1800 g	Sunflower oil	10ml/kg/day	28 days

Salam (Unpublished) [25]	Pakistan	Neonates ≥26 weeks and ≤37 weeks with a birth weight >750g. Gestational age estimated from ultrasound records and Ballard scores at admission	Coconut oil	2 times a day (10 ml/kg/day)	28 days

Sankaranarayanan 2005 [22]	India	Preterm (1500 to 2000g) and term (>2500g)	A. Coconut oil B. Mineral oil	4 times a day	31 days

Soriano 2000 [23]	Brazil	Neonates 28-34 weeks and <1700 g	Soybean oil	3 times a day (12g/kg/day)	30 days

**Figure 2:**
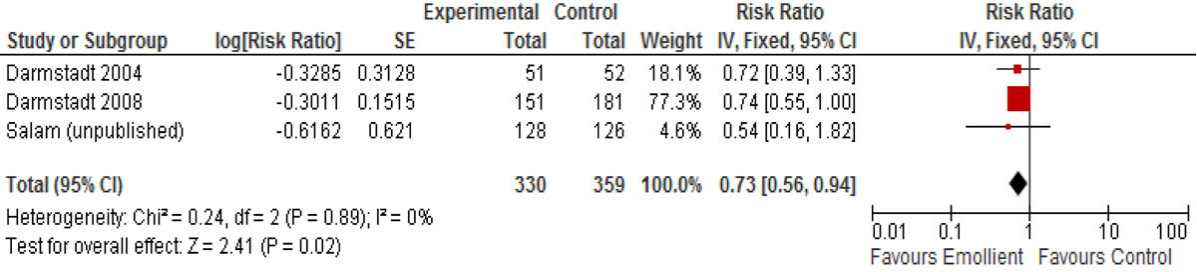
Forest plot for the impact of emollient therapy on neonatal mortality

**Figure 3:**
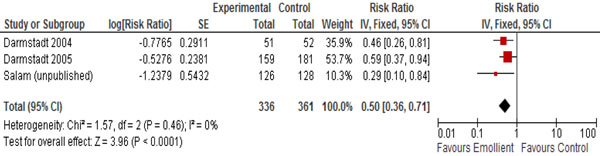
Forest plot for the impact of emollient therapy on hospital acquired infection

**Figure 4:**
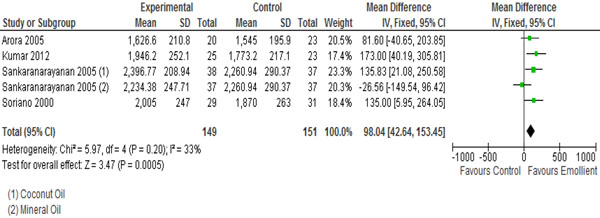
Forest plot for the impact of emollient on weight

In Table 2, we report the quality assessment of studies by outcomes. For neonatal mortality and morbidity, findings were based on three studies; topical emollient therapy significantly reduced neonatal mortality by 27% (RR: 0.73, 95% CI: 0.56, 0.94) (Figure 2) and reduced hospital acquired infections by 50% (RR: 0.50, 95% CI: 0.36, 0.71) (Figure 3). For growth outcomes, data was pooled for four studies; emollient therapy was associated with significant increase in weight (MD: 98.04, 95% CI: 42.64, 153.45) (Figure 4) and weight gain (g/kg/day) (MD: 1.57, 95% CI: 0.79, 2.36). The impacts were non-significant for length (MD: 0.33, 95% CI: -0.15, 0.81) and head circumference (MD: 0.05, 95% CI: -0.30, 0.41).

**Table 2:** Quality assessment by outcome

	Quality assessment					Summary of findings		
				Directness		No of events*		

No of studies	Design	Limitations	Consistency	Generalizability to population of interest	Generalizability to intervention of interest	Intervention	Control	RR / SMD (95% CI)

**Neonatal mortality: *moderate outcome specific quality of evidence***								

Three	RCT	No significant heterogeneity, fixed effect model used	One of the three studies suggest benefit	All studies from the developing countries	Two of the studies used sunflower while one used coconut oil as emollient	04	11	RR: 0.73 [0.56, 0.94]

**Hospital acquired infection: *moderate outcome specific quality of evidence***								

Three	RCT	No significant heterogeneity, fixed effect model used	All studies suggest benefit	All studies from the developing countries	Studies used sunflower and coconut oil	19	33	RR: 0.50 [0.36, 0.71]

**Weight: *moderate outcome specific quality of evidence***								

Four studies (five data sets)	RCT	No significant heterogeneity, fixed effect model used	Three studies suggest benefit	All studies from the developing countries	Studies used coconut, sunflower and soybean oil	149	151	SMD: 98.04 [42.64, 153.45]

**Weight gain (g/kg/day): *moderate outcome specific quality of evidence***								

Two studies (three data sets)	RCT	Significant heterogeneity so a random effect model used	One study suggested benefit	All studies from developing countries	Studies used coconut and sunflower oil	95	97	SMD: 1.57 [0.79, 2.36]

**Length: *moderate outcome specific quality of evidence***								

Two studies (three data sets)	RCT	Significant heterogeneity so a random effect model used	Two studies suggest benefit	All studies from developing countries	Studies used coconut and sunflower oil	124	128	SMD: 0.33 [-0.15, 0.81]

**Head circumference: *moderate outcome specific quality of evidence***								

Two studies (three data sets)	RCT	No significant heterogeneity, fixed effect model used	None of the studies suggest benefit	All studies from developing countries	Studies used coconut and sunflower oil	124	128	SMD: 0.05 [-0.30, 0.41]

### Recommendation for the LiST model

Of the outcomes assessed for effect of topical emollient therapy among preterm newborns in developing countries, we applied the CHERG rules for evidence review to these outcomes. We had data on mortality but since the evidence was weak, we used hospital acquired infection (severe morbidity) as a proxy for neonatal mortality and propose that topical emollient therapy for preterm neonates can reduce neonatal infection related mortality by 50% among preterm neonates <37 weeks gestation.

## Discussion

In this systematic review we estimated the effect of topical emollient therapy on preterm newborns in developing countries. Trials were included that reported various neonatal health outcomes including hospital acquired infection, mortality and anthropometric indices. The studies contributing data in this review were conducted in developing countries including India, Pakistan, Bangladesh, Egypt and Brazil, hence increasing the generalizability of the studies to newborns in LMICs with the highest neonatal mortality and infection rates. All the studies evaluated the impact of topical emollient application in facility settings among preterm neonates. Emollients used in the studies included sunflower, coconut, soybean and mineral oil. The follow-up period ranged from 28-31 days of life. Only one study reported the follow-up till discharge [11].

Topical emollient therapy has been shown to reduce mortality and hospital acquired infections significantly and also improved weight. It was associated with a significantly higher weight gain of 98g than in controls over the first 28 days of life. However the impacts on length and head circumference were non-significant. Neurodevelopment outcomes were reported in only one study and were found to be comparable in the intervention and control group [14]. It is worthwhile to observe that although the evidence is weak for neonatal mortality due to the fewer number of events, the evidence comes from good quality RCTs with no significant heterogeneity reported in the pooled analysis.

The findings from this review contradict with the findings from a published review which indicated increased risk of hospital acquired infection with prophylactic application of topical ointment in preterm neonates [18]. This review differs from our review as it was from studies conducted in HICs with most of the participants being extremely LBW infants and the emollient most commonly used in the trials was aquaphor ointment and most infections were due to coagulase-negative staphylococci. The findings of the previous review cannot be generalized to LMIC settings, as most infections in these settings are due to gram-negative bacteria, for which the attributable morbidity and mortality is much greater than that of coagulase-negative staphylococcal infection [26,27]. Also the commonly used emollients in these settings include coconut, sunflower, almond and olive oil [28].

In this review we have shown that there is potential for benefit of this traditional, simple, affordable, and effective intervention. Efforts should now be concentrated on its scale-up for delivery in hospitals in low-resource settings to reduce neonatal mortality risk due to prematurity and infections. There are no large scale community based effectiveness trials to evaluate its pragmatic benefits or otherwise in community settings. Studies are also required to assess if this intervention is associated with any adverse effects and to explore the benefits of various emollients and to determine which ones are most effective.

## Conclusion

Our analysis of the effect of topical emollient therapy among preterm neonates in developing countries suggests benefit in reducing hospital acquired infections and mortality and improving weight in preterm neonates. However, its implications and feasibility for community scale-up needs to be explored.

## Competing interests

We do not have any financial or non-financial competing interests for this review.

## Authors' contributions

Dr. ZAB was responsible for designing the review and coordinating the review. RAS and JKD were responsible for: data collection, screening the search results, screening retrieved papers against inclusion criteria, appraising quality of papers, abstracting data from papers, entering data into RevMan, analysis and interpretation of data and writing the review. JKD was responsible to apply the GRADE criteria for outcome assessment. ZAB and GD critically reviewed and modified the manuscript.

## Declaration

The publication costs for this supplement were funded by a grant from the Bill & Melinda Gates Foundation to the US Fund for UNICEF (grant 43386 to "Promote evidence-based decision making in designing maternal, neonatal, and child health interventions in low- and middle-income countries”). The Supplement Editor is the principle investigator and lead in the development of the Lives Saved Tool (LiST), supported by grant 43386. He declares that he has no competing interests.

This article has been published as part of *BMC Public Health* Volume 13 Supplement 3, 2013: The Lives Saved Tool in 2013: new capabilities and applications. The full contents of the supplement are available online at http://www.biomedcentral.com/bmcpublichealth/supplements/13/S3.
